# Codon optimality in cancer

**DOI:** 10.1038/s41388-021-02022-x

**Published:** 2021-09-28

**Authors:** Sarah L. Gillen, Joseph A. Waldron, Martin Bushell

**Affiliations:** 1 Cancer Research UK Beatson Institute, Garscube Estate, Switchback Road, Glasgow, G61 1BD UK; 2 Institute of Cancer Sciences, University of Glasgow, Glasgow, UK G61 1QH

**Keywords:** Cancer, Cell proliferation, Translation

## Abstract

A key characteristic of cancer cells is their increased proliferative capacity, which requires elevated levels of protein synthesis. The process of protein synthesis involves the translation of codons within the mRNA coding sequence into a string of amino acids to form a polypeptide chain. As most amino acids are encoded by multiple codons, the nucleotide sequence of a coding region can vary dramatically without altering the polypeptide sequence of the encoded protein. Although mutations that do not alter the final amino acid sequence are often thought of as silent/synonymous, these can still have dramatic effects on protein output. Because each codon has a distinct translation elongation rate and can differentially impact mRNA stability, each codon has a different degree of ‘optimality’ for protein synthesis. Recent data demonstrates that the codon preference of a transcriptome matches the abundance of tRNAs within the cell and that this supply and demand between tRNAs and mRNAs varies between different cell types. The largest observed distinction is between mRNAs encoding proteins associated with proliferation or differentiation. Nevertheless, precisely how codon optimality and tRNA expression levels regulate cell fate decisions and their role in malignancy is not fully understood. This review describes the current mechanistic understanding on codon optimality, its role in malignancy and discusses the potential to target codon optimality therapeutically in the context of cancer.

## Background

The gene expression signature in cancer is often significantly altered, and this is often by elevated activity of oncogenic transcription factors [1, 2]. However, changes at the level of transcription do not always directly reflect the change in protein level; [3] post-transcriptional regulation is also highly important in shaping the cancer proteome. For example, transcript levels are also altered in cancer at the level of mRNA stability [4, 5]. In addition, rapid division and growth of the cancer cells requires increased protein synthesis. To facilitate this, mRNA translation is often dysregulated in tumour cells and the surrounding stroma [6]. Differential expression or covalent modification of a range of translational regulators can enhance the survival and proliferation of the tumour cell clone [7–9] and altered translation has been linked with malignancy due to the upregulation of pro-oncogenic mRNAs to promote proliferation and suppress apoptosis. Overall, due to the increasing evidence for the importance of post-transcriptional regulation in disease, translation is becoming a target area for biomarker discovery and therapeutic intervention [10, 11].

It is the precise sequence composition of each individual mRNA that allows for tight modulation of its translation and stability. The untranslated regions (UTRs) of the mRNA are well-known for their regulatory roles in post-transcriptional regulation [12, 13]. Most of the research on translational regulation in cancer has been centred on regulation exerted by elements within the UTRs. For example within 5ʹUTRs there can be specific sequence motifs such as terminal oligopyrmidine (TOP) motifs, which are important for the mTOR signalling pathway regulation in cancer [14] and highly structured regions in 5ʹUTRs, particularly within oncogenic mRNAs, are reliant on eIF4A for their translation [15–17]. A major example of 3ʹUTR regulation in cancer is through miRNAs that themselves can act as oncogenes or tumour suppressors [18]. Also, 3ʹUTR switching has been noted in proliferating cells [19–23]. The use of alternative 3ʹUTRs can lead to altered translation and/or stability of these messages due to changes in the presence of 3ʹUTR regulatory sites [24]. The significant influence of the coding sequence (CDS) in post-transcriptional control of gene expression has come to light in recent years, particularly differential codon usage, but this regulation in the context of cancer is just beginning.

## The role of codons

The distinguishing feature of the CDS from the UTRs is the presence of information that codes for the protein. A codon dictates which amino acid is to be added to the growing polypeptide during protein synthesis and for most amino acids there are several codons which encode it - these are known as synonymous codons. The existence of synonymous codons allows for tight regulation of protein synthesis, in part, because the translation elongation rates of synonymous codons are not equal [25–27]. An additional layer of regulation pertains to the surrounding codon context of an individual codon—often it is doublets or stretches of certain codon combinations that significantly impact the local translation rate [28]. Differential translation elongation rates can serve multiple purposes beyond just control of protein production levels; this includes: ribosome pausing to allow for mRNA translocation and slowed localised elongation rates to allow for correct protein folding co-translationally [29, 30]. Alternatively, prolonged ribosome pausing at “non-optimal” codons can trigger mRNA destabilisation [31]. The details of these mechanisms will be discussed in a later section.

## tRNA supply and demand

**Fig. 1:**
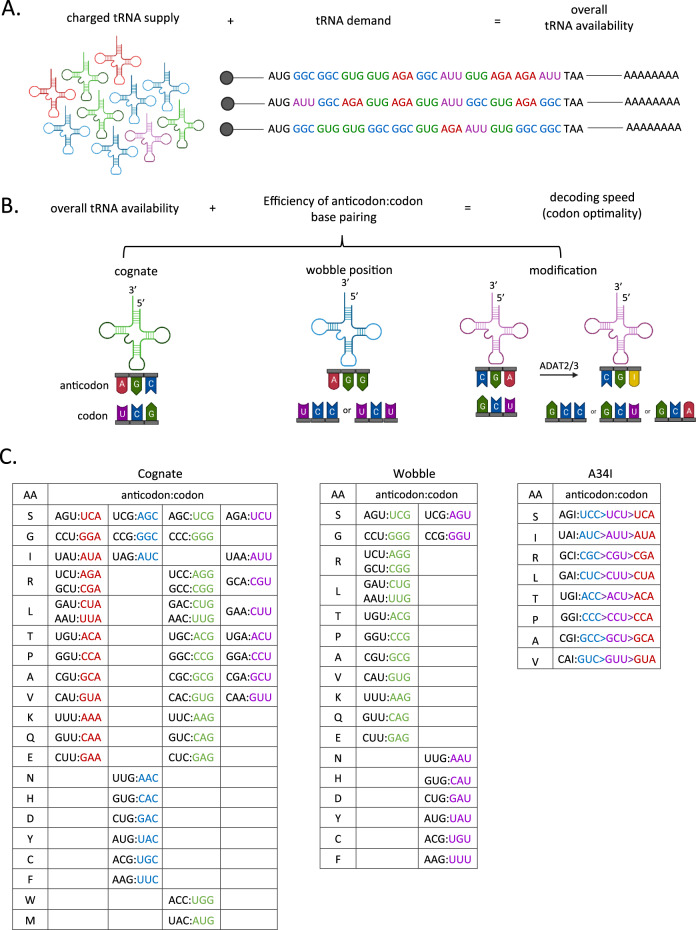
Determinants of codon optimality. **A** The availability of tRNAs is a combination of their supply determined by tRNA gene expression and aminoacylation of the tRNAs, and the demand within the expressed transcriptome for each tRNA. **B** tRNA availability combined with the efficiency of the base-pairing between a given codon and anticodon provides a measure of the optimality of a given codon in a particular cellular context. There are some C-ending codons that require A-to-I modification of the anticodon to be decoded. **C** Tables depict the types of anticodon:codon base pairing that can decode each codon by either cognate, wobble or inosine interactions. Anticodons are in black and are written 3ʹ to 5ʹ. Codons are coloured according to the 3rd nucleotide position and are written 5ʹ to 3ʹ. “>” is used to indicate differences in efficiencies of decoding of inosine containing anticodons with codons that it can base-pair.

A major factor contributing to the decoding rates of specific codons is tRNA availability, both in terms of tRNA abundance and charging with the specific amino acid (Fig. 1A). There are over 400 tRNA genes across the human genome and these encode 46 different tRNA isoacceptors (tRNAs which share the same anticodon but differ in sequence elsewhere in the tRNA) [32]. tRNA isoacceptors use cognate or wobble base pairing to decode 61 codons (Fig. 1B, C) [32]. There are less anti-codons on tRNAs than there are codons, in the majority of cases the first two nucleotides of a codon provide the specificity for a given amino acid and follow standard Watson-Crick base pairing to the tRNA anticodon. Then for certain codons the tRNA ‘wobble position’ is used to recognise the codon, whereby the 5ʹ end of the anticodon (hence 3rd nucleotide of the codon) can still read codons with a non-cognate 3rd nucleotide. For example, the tRNA with anticodon GGA can decode both the UCC and UCU codon (Fig. 1B, C). In addition, modification to the first position of the anticodon can enable additional wobble base-pair interactions. For example, modification of adenosine to inosine (I) on the tRNA increases the number of corresponding codons that can be decoded to include a codon with not just U, but A or C at the third position (Fig. 1B, C). The efficiencies of these base-pairings differs with I:C being the most efficient and I:A the least [33] (Fig. 1C). Also, this wobble position of the tRNA is often subject to a variety of modifications, which influence the ability to base pair with different nucleotides [34, 35]. Codons requiring wobble base-pairing take longer to decode and therefore have increased ribosome dwell times [36, 37].

tRNAs are transcribed by RNA pol III and are processed to form a mature tRNA with modified nucleotides and a 3ʹ CCA tail. The CCA is required for the addition of an amino acid to the tRNA by an aminoacyl-tRNA synthetase and for each specific amino acid there is a different corresponding aminoacyl-tRNA synthetase. The aminoacylated (charged) tRNAs are then delivered to the acceptor site (A-site) of the elongating ribosome by eEF1A. If the tRNA anticodon is complementary to the codon at the A-site the polypeptide chain is extended and the ribosome translocates to the next codon. In general, the expression levels of tRNAs (i.e., tRNA supply) are reflective of the transcriptome expressed—as demonstrated by the fact that the most highly expressed genes are enriched for the most abundantly expressed tRNAs [38]. Interestingly, several investigations have shown specific mRNA and tRNA signatures associated with cellular state [39–41].

## What is “codon optimality”?

**Fig. 2:**
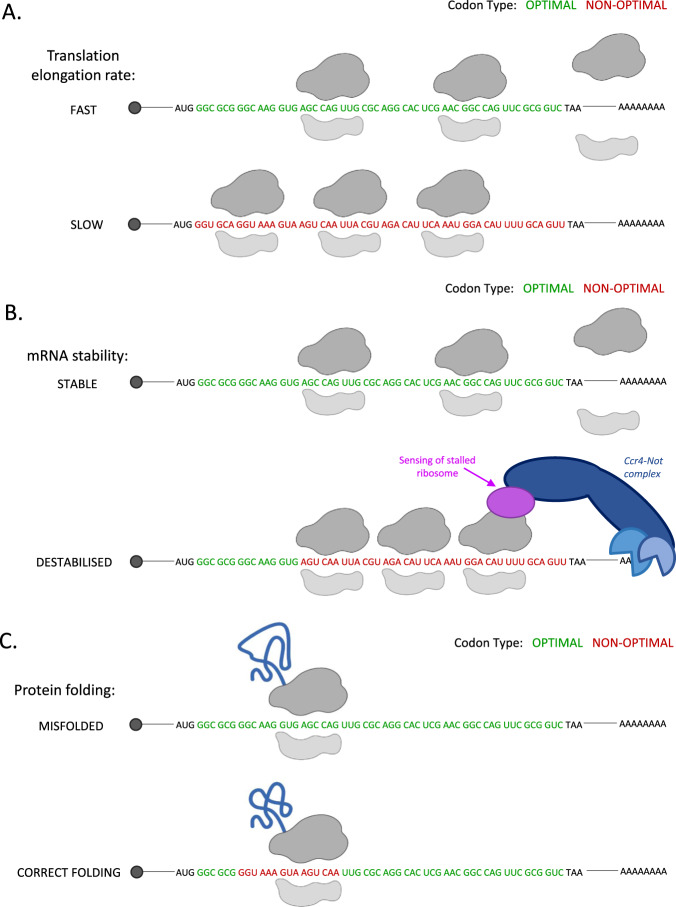
Consequences of codon optimality differences. **A** Elongation rates are greater at optimal codons, leading to increased protein synthesis rates. **B** Stretches of non-optimal codons can lead to a pile-up of paused ribosomes which can trigger recruitment of mRNA decay factors. **C** Short stretches of non-optimal codons can also act to slow translation elongation rates in specific regions to provide time for correct protein folding co-translationally.

Codon optimality is a term broadly used in reference to how the identity of the codons within a given CDS regulate translation elongation rates and mRNA stability and thus overall protein production [42]. In principle, optimal codons are more rapidly decoded meaning the ribosome has shorter dwell times on optimal codons and their presence is associated with increased mRNA stability (Fig. 2). Whereas codons are categorised as non-optimal when the ribosome resides for longer at the codon due to it taking longer for the corresponding tRNA to arrive at the A-site and due to differences in decoding rates of specific tRNA anticodon-codon interactions. ‘Rare’ codons are those which are less frequently utilised with the transcriptome; the tRNAs that recognise them also tend to be lowly expressed. Because of this, rare codons are often non-optimal for protein synthesis, particularly when present in clusters within the CDS. Overall, translation elongation is faster across optimal codons (Fig. 2A), and they are present in stable mRNAs resulting in association with higher protein production from messages enriched for optimal codons.

There are several metrics that can be used to quantify the impact of codons on mRNA translation and stability including: the Codon Adaptive Index (CAI) [43], the tRNA Adaptive Index (tAI) [44] and the Codon Stabilisation Coefficient (CSC) [45]. CAI provides a measure of codon bias in reference to the highly expressed genes, on the assumption that highly expressed genes represent high demand for certain tRNAs due to their elevated expression [43]. This has since been adapted to the tAI which is based on the assumption that tRNA gene copy numbers correlate with tRNA abundance and takes into account the efficiency of the interaction between the codon and the tRNA anticodon ‘wobble’ position [44, 46]. However, both the CAI and tAI are static measures of codon optimality; the Codon Stabilisation Coefficient (CSC) advances these metrics by accounting for mRNA stability dynamics. The CSC for each codon is the Pearson correlation coefficient between the frequency of the codon within the CDS of each mRNA and the mRNA half-live in a given condition, across all mRNAs [45, 47]. For example, a positive CSC would indicate that the frequency of a given codon is associated with greater mRNA stability, whereas a negative CSC would indicate the codon is associated with more rapid mRNA turnover. As the mRNA half-lives determined can vary between cell type, cellular state and because of the techniques used to measure it, the CSC provides a way to relate the codon preference of a given CDS to its translatability in a specific condition.

However, these metrics do not fully take into account the mRNA abundance, given that mRNA expression levels are spread across a large range this would be an additional important aspect to incorporate to obtain more accurate measures of tRNA demand. Also, these metrics tend to assume each codon has a distinct optimality, but the codon context surrounding an individual codon in a message has also been shown to be important for differences in translation rates and mRNA stability [28]. CSCs have been further utilised to try to identify stretches of non-optimal codons within a message [47] and this is an important element to take into consideration when investigating codon optimality.

Hence an “optimal” codon is one for which translation is favoured, compared to a “non-optimal” codon that is less readily decoded. A key determinant of this is tRNA supply; the assumption being that “optimal” codons are those decoded by the most abundant tRNAs [37]. The tRNA supply is also impacted by the extent to which the expressed tRNAs are aminoacylated and the ratio of cognate/near-cognate anticodons for decoding. This supply is balanced by the demand for certain tRNAs within the pool of mRNAs expressed that will impact overall availability of a tRNA in the cell [48]. The translational environment is different depending on the organism, cellular state and different stresses [39, 49, 50]. Thus, codon optimality is not static, the ‘optimality’ of a given codon is dependent on the supply of and demand for translational resources which are condition dependent and currently are difficult to precisely determine.

## Codon usage within functionally related mRNAs

**Fig. 3:**
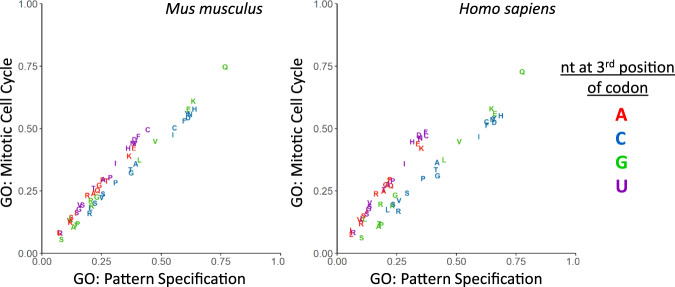
Codon usage differences in proliferation. Synonymous codon usage preferences for mRNAs associated with the GO term: “pattern specification” and ‘mitotic cell cycle’ for mouse (245 and 653 mRNAs respectively) and human (418 and 900 mRNAs respectively). Conducted in a similar manner to Gingold et al. 2014, but with the most recent gene ontology annotation obtained using the R package GO.db. Colours indicate the nucleotide at the 3rd position of the codon and the single letter code is used to indicate the corresponding amino acids.

The codon usage of the human transcriptome has been naturally selected for [51, 52] and synonymous codons are not used equally across the transcriptome [53, 54]. In recent years, research has sought to understand why such bias has evolved. It is now known that functionally associated mRNAs share similar codon usage patterns, particularly those that are co-expressed; [55, 56] and it has been uncovered that distinct codon usage preferences allow for translational regulation of specific gene groups [39, 57, 58]. Specifically, mRNAs that encode proteins that promote proliferation have a highly distinctive codon signature from mRNAs encoding differentiation-promoting proteins [39]. Fig. 3 recapitulates this key finding of Gingold et al. with the most up-to-date gene ontology groups and the codons are coloured by the 3rd nucleotide. This shows in both mouse and human that the synonymous codon usage preference of mRNAs associated with the mitotic cell cycle is bias towards A/U-ending codons (Fig. 3 red & purple), whereas pattern specifying mRNAs have a tendency to contain G/C-ending codons (Fig. 3 blue & green). Several groups have now observed mechanisms that exploit this distinctive codon signature to boost the synthesis of proliferative proteins [39, 57, 59], as detailed in the later sections. This suggests that codon usage can serve as a molecular switch to alter the translational programme to meet the requirements of the cell.

In addition, in yeast, mRNAs encoding functionally related proteins have been shown to have similar mRNA half-lives [45, 60]. Many of these groups contain mRNAs that possess a similar percentage of optimal codons, for example: the glycolysis pathway, pheromone response, tRNA modification and cytosolic ribosomal proteins [45]. This suggests a mechanism whereby codon optimality can be utilised to allow for coordinated regulation of the stability of functionally related groups of mRNAs.

## Mechanisms that coordinate the response to codon optimality

The CDS codon composition and distribution have been highlighted as factors influencing translation rates and mRNA stability in multiple organisms including: E.coli [38, 61], yeast [31, 45, 62, 63], zebrafish [64, 65], mouse [57] and human cells [58, 66–68]. At present the precise mechanisms involved in the sensing of and response to codon optimality within a given mRNA are not fully understood.

### Codon optimality and mRNA stability

The global link between codon optimality and mRNA half-life was first identified in yeast [45]. This study showed that substitution of non-optimal codons to optimal codons in reporter constructs lead to a large increase in mRNA stability [45]. Further research suggests non-optimal codons lead to pausing of the ribosome due to reduced translation elongation rates (Fig. 2A, B) [29]. In yeast it has been suggested that if these pauses are not resolved they may be “sensed”. Dhh1 [31], and Not5 [69] have both been suggested as sensors of codon optimality and other factors may still be discovered that couple this to the triggering of mRNA destabilisation. Dhh1 has been shown to sense non-optimal slowly translated codons and then target these mRNAs for decay (Fig. 2B) [31]. The impact of non-optimal codon stretches on mRNA stability was greatest when they were located towards the 3ʹ end of the CDS. This suggests not only the presence of non-optimal codons, but their positioning within the CDS and the nature of the surrounding codons are also important. Precisely how Dhh1 facilitates this is uncertain, but it has been proposed that Dhh1 interacts with the ribosome [31]. More recently, structural data has shown that the Not5 subunit of the Ccr4-Not complex acts to ‘sense’ codon optimality via a direct interaction with the ribosome [69]. More specifically, Not5 interacts with the E-site of the ribosome that has an unoccupied A-site, presumably due to pausing at non-optimal codons due to their slower decoding rate [69]. The Ccr4-Not complex has also been implicated in codon-mediated regulation of mRNAs in zebrafish [65] and DDX6 (the human homologue of Dhh1) is known to interact with the Ccr4-Not complex via the scaffold protein CNOT1 [70, 71], so perhaps DDX6 acts in concert with the Ccr4-Not complex to link translation elongation to altered mRNA stability.

In mammalian cells DDX6 is involved in directing the translational repression of mRNAs enriched for A/U-ending codons in p-bodies and regulates G/C-rich mRNAs at the level of mRNA stability [72]. This suggests a link not only between codon optimality and mRNA stability, but also translational repression and mRNA storage. Exactly which codons are associated with faster mRNA turnover varies across studies; [45, 68, 72, 73] these differences may be due to the fact that what is an “optimal codon” differs depending on the translational environment—as discussed in the previous section. This concept is demonstrated in a study in *Drosophila* which showed A/U-ending codons were associated with faster mRNA destabilisation in non-neuronal cells, whereas in the neuronal cells it was the G/C-ending codons that were associated with more rapid mRNA decay [74].

Overall, it is clear there is much we still do not understand about how the cell detects codon optimality and its downstream consequences, but there are indicators that the Ccr4-Not complex and its associated helicase DDX6 have a key role in these mechanisms, whether it be by direct interaction with the stalled ribosomes or by interaction with downstream effector proteins.

### The importance of non-optimal codon location and context within the CDS

An additional factor to consider is not only the presence of non-optimal codons, but the positioning of these within the CDS. Codons are not used equally across the CDS [75]. Research shows that across multiple organisms, from *E.coli* to human that the G/C content (and hence codon usage) decreases after the first 25 codons [76]. As detailed in the previous section, reporter assays have shown positional dependency of non-optimal codons on Dhh1-mediated regulation of mRNA half-life [31]. However, although a further global investigation of mRNA half-lives across the yeast transcriptome showed that ribosome pausing occurs at non-optimal codons, it did not observe a link between the position of non-optimal codons within the CDS and mRNA stability [47]. So, whether increased non-optimal codon frequency is sufficient to cause mRNA half-life differences, or if position-dependent effects on mRNA stability are mRNA specific is still unclear.

The surrounding sequence context of an individual non-optimal codon has been identified as a contributing factor to the extent to which non-optimal codons affect translation elongation rate and mRNA stability [28]. It has been shown that stretches of non-optimal codons can be particularly inhibitory to translation [47]. Studies have shown a role for inhibitory codon *pairs* in the regulation of translation and mRNA stability [28, 62, 77]. These pairs consist of non-optimal codons where at least one relies on wobble base-pairing with the tRNA anticodon [28]. In order to be inhibitory, the pair must be consecutive on the CDS and the order of the codons within the pair is also important [28]. In addition, with the specific example of ZEB2 mRNA, stretches of non-optimal codons were shown to impact protein synthesis [78].

While the presence of non-optimal codons can lead to a global reduction in the elongation rate of a message, the purpose can also be to only reduce translation elongation speed in a specific region of the CDS. This serves as an important role in terms of protein structure by ensuring correct protein folding co-translationally and therefore allowing functional protein production [79–81]. Stretches of “slow” codons reduce the local translation elongation rate to provide sufficient time for accurate protein folding (Fig. 2C) [82, 83]. Substitution to “faster” synonymous codons has been shown to increase the likelihood of misfolded proteins, alter protein conformation or lead to protein aggregates [81, 84]. Whether there is a connection between slowed elongation rates for protein folding and ribosome pausing that can trigger mRNA destabilisation pathways has not been investigated.

### Codon optimality interactions with other regulatory features

It is unknown how codon usage interacts with other translational regulatory factors. A recent study demonstrated that miR-430 can promote destabilisation of targets rich in optimal codons [85], suggesting the global impact of codon optimality can be fine-tuned by other additional regulatory mechanisms. miRNAs are often dysregulated in cancer so further investigation of the interplay between different regulators of translation with codon usage could be an interesting area of investigation in the future.

### What about secondary structure?

As non-optimal codons are predominantly described as those with A/U at the wobble position, it should be noted that this changes the propensity for secondary structure within the coding sequence. It is very difficult to unpick differences in translation rate for these codons due to secondary structure or tRNA availability and it could well be a combined effect of both. Work from yeast and *E.coli* proposes that secondary structure in conjunction with tRNA abundance balances translation elongation rates of specific codons [86], in that secondary structure can result in slower translation, but this is overcome by these structured regions often being composed of optimal codons decoded by highly abundant tRNAs. In addition, reporter assays in which both the codon optimality and the propensity of the mRNA to form structure were manipulated demonstrated that increased structure formation in mRNAs with optimal codon usage further stabilised the mRNA [87]. The G/C content of a message also regulates its transcription [88, 89]. So perhaps differences in codon optimality also play a role in determining whether a given message is more highly regulated at the transcriptional and/or translational level.

#### Use of codon optimality to regulate the synthesis of proliferation-promoting proteins

The mRNA and tRNA pools expressed vary across tissues [48, 50, 90]. The tissue-specific mRNA pools differ in their codon usage and these differences have been shown to be conserved between human and mouse [91]. The codon usage preference of tissue-specific mRNAs has been shown to impact translational efficiency by their optimality for the corresponding tRNA availability in the tissue [92], in that their codon usage is most optimal for translation in the tissue they are predominantly expressed in.

The most abundant mRNAs in the cell are enriched in codons that require decoding by the most abundant cognate tRNAs thus ensuring optimal protein synthesis. It has been suggested that in certain cases the opposite is also true, in that certain mRNAs have evolved to specifically have poorly optimised codon usage for ‘normal’ conditions, hence they are poorly expressed when not required by the cell [93]. For example, some circadian clock genes have a greater prevalence of non-optimal codons [55, 94] and cell cycle genes, particularly those that oscillate, are enriched in non-optimal codons, with genes that are expressed in different cell cycle phases showing distinct codon usage [57, 95]. In addition, general levels of tRNAs and tRNA charging enzymes are highest in the G2/M phase, when those proteins most enriched in non-optimal codons are expressed [95]. By sharing a distinct codon preference, groups of genes can be up or downregulated when required, such that under normal conditions they are kept in a translationally repressed state but could be translationally upregulated upon the appropriate signals [93].

### Regulation of “proliferative tRNAs”

**Fig. 4:**
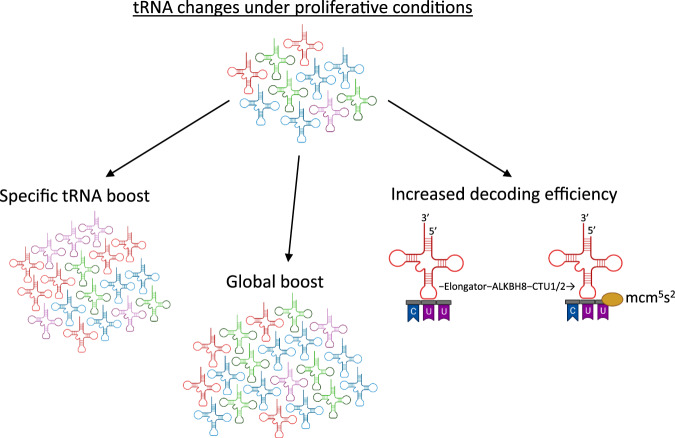
tRNA changes in proliferation. There are several models proposed as to how the translational upregulation of mRNAs preferentially expressed in proliferation occurs. These include a specific increase in the tRNAs that decode A/U-ending codons, a global increase in tRNA expression to overcome limiting elongation rates at non-optimal codons and increased mcm^5^s^2^ U34 modification at the wobble position to increase the elongation rates at specific A-ending codons: CAA, AAA, GAA.

There are multiple hypotheses for how proliferation-associated protein expression is specifically increased. Gingold et al. observe specific upregulation of proliferative mRNAs and their corresponding tRNA supply with large changes in histone modifications around specific tRNA genes, proposing a transcriptional program to coordinate an increase in proliferative mRNA and tRNA expression [39]. Of note, while proliferative mRNAs (enriched for A/U-ending codons) were upregulated in both non-cancerous and cancerous proliferative samples, the mRNA transcriptomes overall more closely clustered based on cell/tissue type as opposed to proliferative status of the samples. This study classified tRNAs as either proliferation- or differentiation-specific tRNAs, and these groupings were highly similar in both cancerous and non-cancerous settings [39]. This model of specific upregulation of proliferative tRNAs to boost translation elongation rates at specific codons enriched in the mRNAs encoding proliferation associated proteins (Fig. 4) is further supported by data showing that the tRNA-ome expressed is reflective of the proliferative status of tissues [48]. Subsequent work has shown that by targeting individual tRNA families with the CRISPR-Cas9 system, most of the identified proliferation-promoting tRNAs were essential in rapidly dividing cells, with the tRNAs associated with differentiation showing higher essentiality in cells with a slower doubling time [96]. Although these differences could be assigned to the proliferative state of the cell, it seemed that the cellular origin was also an important factor [96].

An alternative model has been proposed by Guimaraes et al. that rather than specific upregulation of ‘proliferative tRNAs’, there is a global increase in all tRNA levels in proliferative conditions (Fig. 4), due to the overall boost in translation required in highly proliferative cells. This model is proposed because they found no differential tRNA expression between cells grown in 10% serum compared with 1% but did observe increased translational efficiency on mRNAs enriched in A/U-ending codons [57]. A global increase in tRNA levels then overcomes the reduced elongation rates of proliferation specific mRNAs that are limited by their enrichment for A/U-ending codons that have ‘rare’ tRNA anticodons relative to the general tRNA supply [57].

It is probable that each study has revealed a pathway that cells can use to boost protein synthesis from mRNAs that are non-optimal for translation under normal conditions. The commonality between the models is that tRNA supply for decoding of non-optimal codons is a limiting factor to be overcome in order to facilitate the increased protein synthesis of proliferation-specific mRNAs. While the increased mRNA-level of these proliferation-specific mRNAs may in part be explained by increased transcription, the increased translation elongation rates at these A/U-ending codons in proliferative conditions also likely stabilises these proliferative mRNAs.

## tRNAs in cancer

Increased proliferation requires increased protein synthesis to facilitate this boost in mRNA translation, the tRNA supply needs to also increase – this can be global or specific upregulation of tRNAs. tRNAs are transcribed by RNA polymerase III and increased RNA Pol III activity has long been associated with cancer [97], as are elevated tRNA levels [98]. This is in part due to key oncogenes such as MYC, or pathways that are dysregulated in cancer like mTORC1 signalling, impacting on RNA pol III and consequently altering tRNA biogenesis [99, 100]. In combination an upregulation of tRNA synthetases, tRNA modification enzymes and other translation factors often occurs in cancer which together support the translational enhancement required for rapid proliferation [41].

### Altered tRNA-ome

The first attempt at exploring tRNA expression in cancer tissue showed that both nuclear and mitochondrial encoded tRNAs were upregulated both globally and specifically in tumour compared to normal breast tissue [101]. The relative differential expression of tRNA levels was observed both at the amino acid and isoacceptor level. tRNAs encoding both polar and charged amino acids showed the highest relative increase in cancer tissue [101]. They also compared the tRNA profiles to the codon usage of either cancer specific, cell line specific or house-keeping genes and found that only the cancer specific genes were enriched in the codons decoded by the tRNAs with greater expression in the cancer tissues. A global upregulation of tRNAs in cancerous cells was also found by Mahlab *et al*., but this study did not identify any relative changes in specific tRNAs [98].

There are two studies that have utilised The Cancer Genome Atlas (TCGA) database to assess tRNA expression for many samples across cancer types. These identify several tRNAs with altered expression in tumorigenesis [41, 48]. Between paired normal and tumour samples the greatest differences in tRNA expression at the amino acid level were for tRNAs that decode arginine, cysteine and valine [41]. This study also noted that certain tRNAs were upregulated in some cancers and downregulated in others [41], indicating cancer specific alterations, yet most cancers showed a greater number of upregulated tRNAs than downregulated tRNAs, suggesting that overall tRNA expression is increased in cancer. High tRNA-Arg^UCG^ and tRNA-Arg^UCU^ expression were equated with poor survival in kidney renal clear cell carcinoma, whereas it was low levels of tRNA-Thr^UGG^ and tRNA-Pro^UGU^ associated with poor survival [41].

Increased supply of specific tRNAs has also been demonstrated to facilitate metastasis [40]. Overexpression of both tRNA-Glu^UUC^ and and tRNA-Arg^CCG^ is observed in metastatic breast cancer cell lines compared to their non-metastatic parental lines. By either overexpressing these tRNAs in the non-metastatic lines or knocking them down in the metastatic cell lines, this study showed that the expression levels of these tRNAs is correlated with the metastatic potential [40]. EXOSC2 and GRIPAP1 were identified as key downstream targets of tRNA-Glu^UUC^ and their increased expression, at least in part, explains the increased metastatic potential following the upregulation of tRNA-Glu^UUC^ [40].

A lack of correlation between cellular state and differential tRNA expression has been noted albeit using an indirect measurement of tRNA levels [102], however the ability of a global upregulation of tRNA expression to specifically impact the translation rates of a subset of mRNAs has also been observed [57]. In addition, it is not just changes in tRNA expression, but also tRNA modification differences that have been observed in cancer [103]. Differences in tRNA modification profiles have been observed between paired fast and slow growing cells and between endometrial cancer tissue and their adjacent normal tissues, with most modifications increasing in the more proliferative cells [104]. In addition, adenosine to inosine editing at the wobble position (A34I) of tRNAs was shown to be higher in self-renewing stem cells compared with differentiating cells [105]. This led to increased decoding of NNC and NNU codons that utilise A34I edited tRNAs. However, whether this leads to increased protein expression of mRNAs enriched for these codons remains to be shown. Furthermore, the RNA methyltransferase NSUN2 has been shown to be associated with metastatic progression in breast cancer [106] and poor survival in Head and Neck Squamous Carcinoma [107]. However increased modification is not always the case, eighteen types of tRNA modifications and seven tRNA-modifying genes are downregulated in non-small lung cancer [108] and human tRNA methyltransferase 9-like (hTRM9L), which catalyses tRNA wobble base modifications has been shown to be down-regulated in several cancers [109].

The mcm^5^s^2^ modification of U34 promotes decoding of AAA, GAA, and CAA codons and is carried out by a cascade of enzymes, including the Elongator complex [103]. Several components of the elongator complex have been shown to be upregulated in breast cancer tissue [110] and melanoma [111]. Also, ablation of the catalytic subunit elp3, reduced tumour growth in wnt dependent intestinal tumours [112] and impaired metastasis in an invasive mouse model of breast cancer [110] and re-sensitised resistant BRAF^V600E^ melanoma cells to a BRAF^V600E^ inhibitor [111]. These studies provide evidence that U34 modification of tRNAs is required for the decoding of specific mRNAs involved in tumour initiation, invasion and therapeutic resistance, such as SOX9 [112], DEK [110] and HIF1α [111] and HNRNPQ [113], due to the enrichment of these mRNAs in AAA, GAA, and CAA codons. mcm^5^s^2^U34 modifications therefore provide a mechanism to increase the decoding rate of mRNAs that are enriched in these specific A-ending codons, that are often associated with proliferative mRNAs, without increasing the abundance of overall or specific tRNAs (Fig. 4). In addition, transcriptome changes that occur following ERα depletion are translationally offset in mRNAs specifically enriched for codons that can utilise mcm^5^s^2^U34 modification decoding and ERα regulates the expression of enzymes involved in mcm^5^s^2^U34 modification in prostate and breast cancer cells [114]. This suggests the regulation of codon optimality and thus decoding rates by mcm^5^s^2^U34 modification may also play an important role in translational buffering allowing protein levels to be maintained despite transcriptional changes.

### tRNAiMet

Of all tRNAs, the one unique tRNA, which is presumably equally required by all mRNAs is of course the initiator methione tRNA_i_^Met^. Upregulation of tRNA_i_^Met^, but not tRNA_e_^Met^ has been observed in proliferative samples [39] and overexpression of tRNA_i_^Met^ in non-transformed breast cell lines lead to increased cellular metabolism and proliferation [101]. tRNA_i_^Met^ overexpression also altered the expression of additional tRNAs, suggesting a possible feedback mechanism between tRNA levels and tRNA transcription [101], whether this feedback is more widely applicable to overexpression of other specific tRNAs is unknown. A more specific role for tRNA_i_^Met^ has also been identified in promoting tumorigenesis through enhancing the cancer associated secretome, resulting in increased extracellular matrix deposition [115]. This is further supported by ribosome profiling data showing the specific translational upregulation of extracellular matrix proteins in hepatocellular carcinoma [116].

Overall, an increase in tRNA_i_^Met^ could help overcome the main rate-limiting step in protein synthesis: translation initiation, which could explain why there is a global boost in translation following tRNA_i_^Met^ overexpression. In these circumstances it could be that the influence of codon optimality on translation rates of certain mRNAs increases as the rate limiting step shifts from translation initiation to translation elongation and hence the tRNA supply and demand become of even greater importance than in a ‘normal’ situation.

## Oncogenes/tumour suppressor genes

One way in which oncogenes support rapid cell growth is by upregulating the expression of components of the translational machinery to enable increased levels of protein synthesis. For example, C-MYC increases the transcription of ribosomal proteins, ribosomal RNA, and mRNA cap binding complex components [117–119]. Both the oncogene C-MYC and the tumour suppressor’s p53 and Rb bind TFIIIB activating and repressing RNA polymerase III transcription respectively [99, 117, 120]. Therefore, dysregulation of these protein leads to altered tRNA supply in cancer which may not only be on a total tRNA-level, but also changes in specific tRNA levels [121]. As there are often major transcriptional changes associated with oncogene/tumour suppressor expression changes in cancer it is likely they also alter the tRNA demand by re-sculpting the transcriptome expressed.

### Synonymous mutations

Synonymous mutations were once considered silent as they do not alter the amino acid sequence of the protein. However, it has become clear that synonymous mutations can still impact protein synthesis and function [94, 122]. A large-scale study of mutations across cancers [123] identified 23.4% (659,154 mutations) of pan-cancer point mutations as synonymous and 26.8% of these were seen consistently across tumour types. This study showed that these recurrent synonymous mutations are more prevalent in tumours that have a lower mutational load suggesting they are particularly specific mutations. Studies have shown that the most frequent form of synonymous mutation is C/G - > T/A [123, 124]. A recent study using ribosome profiling data from patient liver cancer samples identifies synonymous mutations that change codon optimality significantly impact translation rates at these specific codons [116, 125]. Synonymous mutations to G/C led to a reduction in A-site ribosome occupancy at these specific positions when comparing the occupancy on the wild-type allele to the mutated allele within tumour samples, whereas for changes to A/U there was an increase in A-site ribosome occupancy [125]. Whether this is sufficient to alter protein levels was not examined, but this does demonstrate how single synonymous mutations can directly impact local translation elongation rates.

Li et al. showed oncogenes had a greater translational efficiency and tumour suppressors lower translational efficiency in tumour samples compared to normal tissue. This group together with others have observed an enrichment for synonymous mutations to optimal codons in oncogenes, and to non-optimal codons within tumour suppressors genes [125–127]. Suggesting these mutations could serve to increase the synthesis of oncogenic proteins or decrease the synthesis of tumour suppressors thus driving the initiation of cancer development. Interestingly, the prevalence of synonymous mutations in particular oncogenes varies across tissues [123]. Given that the tRNA-ome also varies across tissues [90] it may be that certain synonymous mutations are more influential on cancer development depending on the cell origin. The localisation of synonymous mutations within the CDS of oncogenes is not random, in that they are more prevalent in specific regions [123]. Synonymous (and missense) mutations are more likely to occur in conserved regions which is likely why they can be so impactful on protein function.

### Codon signatures

While it is clear that, in general, mRNAs associated with proliferation have a distinct codon usage from mRNAs associated with differentiation, what does this look like at the individual mRNA level within oncogenes and tumour suppressors?

One of the best studied oncogene families in terms of codon optimality is the RAS family of small GTPases, composed of KRAS, HRAS and NRAS. Despite these proteins sharing ~85% identity at the amino acid level, they share only ~15% codon identity, with KRAS being enriched in proliferation associated A/U ending codons, often referred to as “rare” codons, and HRAS being enriched in the more commonly found G/C ending codons and NRAS being intermediate [128]. The enrichment of rare codons in KRAS has been shown to limit its translation, which is relieved when these codons were converted to more common G/C ending codons in cultured mammalian cells [128–130]. In addition to altered translation rates, the codon usage of KRAS affected its mRNA abundance, whether this occurs at the level of transcription [129] or mRNA stability [130] is debated.

The expression level of RAS has previously been shown to impact tumorigenesis, in that high levels lead to senescence, whereas lower levels result in tumour development [131]. Interestingly, after heterozygous conversion of 27 of the KRAS rare codons to more optimal codons, KRAS levels are elevated and this is accompanied by reduced tumour formation and senescence induction [132]. Thus, suggesting that the increased prevalence of non-optimal codons in KRAS functions to maintain its low expression levels and therefore prevent the induction of cellular senescence. However, given higher expression of oncogenic KRAS drives increased tumour growth in transformed cells [128], it seems that a mechanism to increase its protein synthesis would be required once the barrier of oncogene induced senescence is overcome. One mechanism could be that the altered tRNA-ome in proliferative conditions allows for faster decoding of A/U-ending codons, which could increase KRAS translation and/or mRNA stability. In support of this, reporter studies have shown that the wild-type KRAS was upregulated at both the protein and RNA level more so than a codon optimised KRAS reporter in fed vs starved conditions [130].

The utilisation of synonymous codon usage to differentially regulate the expression levels of oncogenes between normal and proliferative conditions, could be a more widespread mechanism by which oncogenes drive tumorigenesis. Data to support this includes the observation that several cancer-related protein families have high amino acid identity but the most frequently mutated member in cancer is most enriched in proliferation associated codons [130].

## Conclusions and future perspectives

The presence of synonymous codons within the genetic code has enabled a mechanism to develop which allows regulation at the mRNA decoding and stability level, whether it is to optimise translation of essential proteins, regulate localised translation rates to ensure correct co-translational protein folding or to up/down regulate specific gene sets when required. The fact that elongation can be directly sensed in this manner, means that elongation rates will contribute to the overall protein expression, even for the majority of mRNAs in which initiation is the rate limiting step of translation. However, there remains inconsistencies in our understanding of the exact interplay between tRNA levels, tRNA modification, elongation rates and codon optimality, particularly in higher eukaryotes. Also, there is still much to be understood about how codon optimality interacts with other factors to regulate mRNA stability and protein output. For example, although loss of wobble U34 modification leads to the specific elongation block at NAA codons, this is not sufficient for a reduction in protein expression levels. Rather, those proteins that decrease in expression also possess a pentameric hydrophilic motif which causes the synthesised proteins to aggregate and be subsequently degraded [133]. One possible explanation for this could be that altered elongation rates at NAA codons lead to protein misfolding co-translationally and thus expose this motif.

Codon optimality is dynamic, and dependent on a multitude of factors that shape the translational environment. Specific functionally related gene sets have acquired a similar pattern of codon usage that is non-optimal for ‘normal’ conditions, but by increased expression of tRNA subsets or alterations to tRNA modification pathways, the optimality of these codons can effectively be switched on. This leads to specific codons being optimal for mRNA translation/stability depending on the cellular state, cell cycle phase and other influences such as stress.

Until recently, tRNAs have been inherently difficult to sequence because they are highly structured and heavily modified, but now methods have been developed to overcome these limitations and allow us to ascertain the tRNA-ome globally, enabling the interrogation of differential tRNA pools between conditions. To overcome the major tRNA modifications that would otherwise block the reverse transcription step of library preparation, these modifications are typically either removed, such as by demethylation with AlkB treatment [134] or alternatively a highly processive reverse transcriptase, named TGIRT, is used that can read-through modifications with a high error rate, to provide full-length tRNA reads regardless of tRNA modification status [135, 136]. Further improvements also include the use of custom ligation methods to add sequencing adapters despite problems due to tRNA structure and some methods also assess the tRNA charging [137].As described in the earlier sections, these methods have enabled the identification of differential tRNA expression in proliferation compared to differentiation. Whether it is indeed specific upregulation of the proliferative tRNAs or global upregulation, either would lead to increased expression of proliferative mRNAs. In the future, it might be possible to therapeutically manipulate the tRNA pools to reduce proliferation-specific protein synthesis and therefore halt tumour development. Whichever mechanism is at play there are still unanswered questions about the precise details – what factors are driving the increase in proliferative mRNA and tRNA expression? What factors direct upregulation of a specific subset of tRNAs? In addition, there is still much to understand about the regulation of tRNA modifications and how this impacts codon optimality in a condition specific manner. Moreover, most tRNA studies have assessed the tRNA-level, but do not always identify the charging status of the tRNA, so there may be further regulation at the level of tRNA charging.

Developments in next generation sequencing techniques not only allow us to identify the translating mRNA population (polysome profiling), but now enable nucleotide resolution of ribosome occupancy for each transcript (ribosome profiling [138],) meaning that ribosome residency at each codon of a message can be examined. This could be used in combination with improved tRNA-seq methods to accurately determine changes in tRNA abundance and charging. These complementary techniques provide a very powerful approach in which to dissect the underlying mechanisms at play and hence provide a global view of the dynamics of codon optimality differences in cancer. However, it is important to note that while ribosome occupancy generally correlates with protein synthesis rates, its increase can also indicate ribosome stalls and slowed elongation. Therefore, given that changes in tRNA levels and/or codon optimality will lead to changes in the rate of elongation, an improved approach is to combine ribosome profiling with pulsed SILAC [139] to provide a direct measure of changes in protein synthesis rate. This could also be used to identify potential pause sites that are resolved or augmented in different conditions and could be used to investigate the regulation that may be occurring at specific rare/non-optimal codons in mRNAs encoding oncogenes/tumour suppressors. Thus, we can now aim to understand the role of codons in cancer at nucleotide resolution allowing us to study the dysregulation of translation in key oncogene/tumour suppressor mouse models.

Since increased cell proliferation is such a key characteristic of cancer, it will be interesting to explore how the distinguishing proliferative codon usage signature can be utilised in the specific targeting of cancer cells and whether specific tRNAs or indeed amino acids could be targeted to exploit any potential vulnerabilities. Furthermore, given that different tissues have different codon usage patterns in the specific transcriptome expressed, the fact that viruses adapt their codon usage to the tissue they infect, and mRNA-based therapeutics are a possible future treatment for cancer, understanding codon optimality in cancer could be important in the design of potential future mRNA-based therapeutics to maximise expression in the target cell populations.
